# Two new corticioid species of Phanerochaetaceae (Polyporales, Basidiomycota) from Southwest China

**DOI:** 10.3389/fcimb.2023.1105918

**Published:** 2023-02-03

**Authors:** Qiu-Yue Zhang, Zhan-Bo Liu, Hong-Gao Liu, Jing Si

**Affiliations:** ^1^ Institute of Microbiology, School of Ecology and Nature Conservation, Beijing Forestry University, Beijing, China; ^2^ Faculty of Agronomy and Life Sciences, Zhaotong University, Zhaotong, Yunnan, China

**Keywords:** new taxa, phlebioid clade, phylogeny, taxonomy, wood-decaying fungi

## Abstract

Two new corticioid fungi in the family Phanerochaetaceae, *Phanerochaete shenghuaii* and *Rhizochaete variegata*, are described and illustrated from Southwest China based on morphological characteristics and molecular data. *Phanerochaete shenghuaii* is characterized by annual, effused, inseparable basidiocarps from substrate, ivory white to cream hymenial surface when juvenile, buff to yellowish brown with age, buff in KOH, a monomitic hyphal system, smooth cystidia, and ellipsoid basidiospores measuring 4.8–6 × 2.5–3.8 µm. *Rhizochaete variegata* is characterized by annual, effused, easily separable basidiocarps from substrate, buff-yellow to clay-pink fresh hymenial surface becoming cream to buff upon drying, violet in KOH, a monomitic hyphal system, encrusted cystidia, and ellipsoid basidiospores measuring 3–4 × 2.2–3 µm. The phylogenetic analyses based on ITS + nLSU rDNA sequences confirm the placement of the two new species, respectively, in the *Phanerochaete* clade and the *Rhizochaete* clade of Phanerochaetaceae. Phylogenetically related and morphologically similar species to these two new species are discussed.

## Introduction

A phlebioid clade is a large group of Polyporales, comprising three families (Phanerochaetaceae Jülich, Irpicaceae Spirin & Zmitr., and Meruliaceae Rea), which accommodates massive corticioid fungi (Wu et al., 2010; Dai, 2011; Justo et al., 2017; He et al., 2019). Most members of the phlebioid clade are saprotrophs on dead wood, causing white rot, which plays an essential role in the maintenance of forest ecosystems (Justo et al., 2017; Ryvarden and Melo, 2017). However, compared with the antrodia and core polyporoid fungi in Polyporales, the phlebioid clade, especially corticioid fungi, has not been intensively studied, with some corticioid genera being known as paraphyletic or polyphyletic, and their members are scattered in different lineages, not fully consistent with the morphological features (Ortiz-Santana et al., 2013; Justo et al., 2017; Cui et al., 2019).

*Phanerochaete* P. Karst., established based on *P. velutina* (DC.) P. Karst., is the largest corticioid genus with more than 100 described species in Phanerochaetaceae (Burdsall, 1985; Kirk et al., 2008; Wu et al., 2010; Ghobad-Nejhad et al., 2015). The genus has a worldwide distribution and is characterized by white-rot, resupinate, and membranaceous basidiocarps; smooth or tuberculate hymenial surface; a monomitic hyphal system; generative hyphae mostly simple septate; the presence of smooth or encrusted cystidia; and thin-walled, non-amyloid, and acyanophilous basidiospores (Wu, 2000; Wu et al., 2010; Floudas and Hibbett, 2015; Ghobad-Nejhad et al., 2015). The diversity and taxonomy of *Phanerochaete s.l.* in China have been studied for 30 years (Wu, 1990; Wu, 1995; Wu, 1998; Wu, 2000; Wu, 2004; Wu, 2007; Xiong and Dai, 2009; Wu et al., 2010; Ghobad-Nejhad et al., 2015; Liu and He, 2016; Chen et al., 2018; Wu et al., 2018a; Wu et al., 2018b). Early studies focused on fungi of Taiwan Province and were mostly based solely on morphology. Recent studies have confirmed that the genus is highly polyphyletic and its species are distributed throughout the phlebioid clade, comprising a number of *Phanerochaete* species assembled in a highly supported clade, referred to as the core *Phanerochaete* clade, containing the type *P. velutina* (Wu et al., 2010; Floudas and Hibbett, 2015; Justo et al., 2017; Chen et al., 2021).

*Rhizochaete* is a small genus introduced by Greslebin et al. (2004), based on *R. brunnea* Gresl. et al., as a segregate of *Phanerochaete*, differing mainly by the reaction of basidiocarps and rhizomorphs (hyphal cords) with KOH: basidiocarps of *Rhizochaete* become red or violet in KOH, while they keep unchanged in *Phanerochaete*. *Rhizochaete* is characterized by resupinate, loosely adnate basidiocarps, with smooth to tuberculate hymenophore, usually turning red to violet in KOH, a monomitic hyphal system with simple septa or clamp connections, cylindrical to ellipsoid basidiospores, usually non-amyloid and acyanophilous (Nakasone et al., 2017; Gu and Zhao, 2021). Since *Rhizochaete* was erected, the number of newly named species is increasing continuously. Based on studying the parenthesome structure of some corticioid fungi, Bianchinotti et al. (2005) reported that three *Rhizochaete* species had perforate septal dolipore caps or parenthesomes. Nakasone et al. (2017) described a new species of *Rhizochaete* from Belize and transferred three additional species to the genus based on morphological and molecular data. Gu and Zhao (2021) reported two new species based on a combination of morphological features and molecular evidence. So far, approximately 17 species have been accepted in *Rhizochaete* worldwide (Greslebin et al., 2004; Chikowski et al., 2016; Nakasone et al., 2017; Gu and Zhao, 2021). Recently, a family-level classification of Polyporales or phlebioid fungi has shown that the genus *Rhizochaete* nested within Phanerochaetaceae, grouped with *Hapalopilus* P. Karst., *Phaeophlebiopsis* Floudas & Hibbett, and *Phlebiopsis* Jülich (Greslebin et al., 2004; Wu et al., 2010; Ghobad-Nejhad et al., 2015; Chen et al., 2021; Zhao et al., 2021).

During investigations on the diversity of wood-rotting fungi from China, four unknown corticioid specimens were collected from Southwest China, and their morphology corresponded to the concepts of *Phanerochaete* and *Rhizochaete*. To confirm their affinity, phylogenetic analyses based on the internal transcribed spacer (ITS) and nLSU rDNA sequences were carried out. Both morphological characteristics and molecular evidence demonstrated that these four corticioid specimens represent two new species of Phanerochaetaceae. So, we describe them in the present paper.

## Materials and methods

### Morphological studies

The studied specimens are deposited in the herbarium of the Institute of Microbiology, Beijing Forestry University (BJFC). Macro-morphological descriptions are based on field notes and measurements of herbarium specimens. Micro-morphological data and drawings are obtained from the dried specimens and observed under a light microscope following Chen et al. (2021) and Wu et al. (2022b). Color terms followed Petersen (1996). Sections were studied at a magnification up to ×1,000 using a Nikon Eclipse 80i microscope with phase contrast illumination (Nikon, Tokyo, Japan). Drawings were made with the aid of a drawing tube. Microscopic features, measurements, and drawings were made from slide preparations stained with Cotton Blue and Melzer’s reagent. Basidiospores were measured from sections cut from the hymenophore. To present the variation of basidiospores size, 5% of measurements were excluded from each end of the range and are given in parentheses. The following abbreviations are used: IKI = Melzer’s reagent; IKI− = neither amyloid nor dextrinoid; KOH = 5% potassium hydroxide; CB = Cotton Blue; CB− = acyanophilous; *L* = arithmetic average of all basidiospores length; *W* = arithmetic average of all basidiospores width; *Q* = variation in the *L*/*W* ratios between the specimens studied, (*n* = *x*/*y*) = the number of basidiospores (*x*) measured from a given number of specimens (*y*).

### DNA extraction and sequencing

A cetyltrimethylammonium bromide (CTAB) rapid plant genome extraction kit (Aidlab Biotechnologies, Co., Ltd., Beijing, China) was used to extract DNA (Wu et al., 2020). The following primer pairs were used to amplify the DNA: ITS5 (5′‐GGA AGT AAA AGT CGT AAC AAG G‐3′) and ITS4 (5′‐TCC TCC GCT TAT TGATAT GC‐3′) for the ITS regions (White et al., 1990); LR0R (5′‐ACC CGC TGA ACT TAA GC‐3′) and LR7 (5′‐TAC TAC CAC CAA GAT CT‐3′) for nuclear large subunit rDNA (nLSU) (Vilgalys and Hester, 1990). The PCR products were purified with a Gel Extraction and PCR Purification Combo Kit (Spin-column) at Beijing Genomics Institute (BGI), China. The purified products were then sequenced on an ABI-3730-XL DNA Analyzer (Applied Biosystems, Foster City, CA, USA) using the same primers as in the original PCR amplifications. All newly generated sequences were submitted to GenBank and are listed in **Table 1**.

**Table 1:** Taxa information and GenBank accession numbers of sequences used in this study.

Species	Specimen no.	Locality	ITS	nLSU	Literature
*Bjerkandera adusta*	HHB-12826-Sp	Alaska, United States	KP134983	KP135198	Justo et al. (2017)
*B. centroamericana*	L-13104-sp	Costa Rica	KY948791	KY948855	Wu et al. (2010)
*Hapalopilus eupatorii*	Dammrich 10744	Germany	KX752620	KX752620	Miettinen et al. (2016)
*H. nidulans*	JV0206/2	Sweden	KX752623	KX752623	Miettinen et al. (2016)
*H. percoctus*	Miettinen 2008	Botswana	KX752597	KX752597	Miettinen et al. (2016)
*Phaeophlebiopsis caribbeana*	HHB-6990	United States	KP135415	KP135243	Floudas and Hibbett (2015)
*P. himalayensis*	He 3854	Hainan, China	MT386378	MT447410	Zhao et al. (2021)
*P. peniophoroides*	FP-150577	United States	KP135417	KP135273	Floudas and Hibbett (2015)
*P. ravenelii*	CBS 411.5	France	MH856691	MH868208	Vu et al. (2019)
*P. ravenelii*	FCUG 2216	France	–	GQ470674	Wu et al. (2010)
*Phanerochaete aculeata*	GC 1703-117	Taiwan, China	MZ422785	MZ637177	Chen et al. (2021)
*P. aculeata*	Wu 880701-2	Taiwan, China	MZ422787	GQ470636	Chen et al. (2021)
*P. albida*	GC 1407-14	Taiwan, China	MZ422788	MZ637179	Chen et al. (2021)
*P. albida*	WEI 18-365	Taiwan, China	MZ422789	MZ637180	Chen et al. (2021)
*P. allantospora*	KKN-111-Sp	Arizona, United States	KP135038	KP135238	Chen et al. (2021)
*P. allantospora*	RLG-10478^*^	Arizona, United States	KP135039	–	Chen et al. (2021)
*P. alnea*	Larsson 12054 (GB)	Norway	KX538924	–	Floudas and Hibbett (2015)
*P. alnea*	FP-151125	Michigan, United States	KP135177	MZ637181	Spirin et al. (2017)
*P. alnea* ssp. *lubrica*	Spirin 8229	Washington, United States	KU893876	–	Floudas and Hibbett (2015)
*P. alnea* ssp*. lubrica*	HHB-13753	Alaska, United States	KP135178	–	Spirin et al. (2017)
*P. alpina*	Wu 1308-61^*^	Yunnan, China	MZ422790	MZ637182	Chen et al. (2021)
*P. alpina*	Wu 1308-77	Yunnan, China	MZ422791	MZ637183	Chen et al. (2021)
*P. arizonica*	RLG-10248-Sp	United States	KP135170	KP135239	Floudas and Hibbett (2015)
*P. australis*	GC 1704-27	Taiwan, China	MZ422793	MZ637185	Floudas and Hibbett (2015)
*P. australis*	HHB-7105-Sp	United States	KP135081	KP135240	Floudas and Hibbett (2015)
*P. australosanguinea*	MA-Fungi 91308	Chile	MH233925	MH233928	Phookamsak et al. (2019)
*P. australosanguinea*	MA-Fungi 91309^*^	Chile	MH233926	MH233929	Phookamsak et al. (2019)
*P. bambusicola*	Wu 0707-2	Taiwan, China	MF399404	MF399395	Wu et al. (2018b)
*P. brunnea*	He 1873	Zhejiang, China	KX212220	KX212224	Liu and He (2016)
*P. burdsallii*	FP-101018-sp	Minnesota, United States	AY219348	–	Liu and He (2016)
*P. burdsallii*	He 2066^*^	Wisconsin, United States	MT235690	MT248177	de Koker et al. (2003)
*P. burtii*	FD-171	Massachusetts, United States	KP135116	–	Floudas and Hibbett (2015)
*P. burtii*	HHB-4618-Sp	United States	KP135117	KP135241	Floudas and Hibbett (2015)
*P. calotricha*	Vanhanen-382	Finland	KP135107	–	Floudas and Hibbett (2015)
*P. canobrunnea*	CHWC 1506-66	Taiwan, China	LC412095	LC412104	Wu et al. (2018a)
*P. canolutea*	Wu 9712-18	Taiwan, China	MZ422796	–	Chen et al. (2021)
*P. canolutea*	Wu 9211-105^*^	Taiwan, China	MZ422795	GQ470641	Chen et al. (2021)
*P. carnosa*	HHB-9195	United States	KP135129	KP135242	Floudas and Hibbett (2015)
*P. chrysosporium*	HHB-6251-Sp	United States	KP135094	KP135246	Floudas and Hibbett (2015)
*P. chrysosporium*	PC139	Taiwan, China	MZ422797	MZ637186	Floudas and Hibbett (2015)
*P. cinerea*	He 5998^*^	Hainan, China	–	MT248171	Xu et al. (2020)
*P. cinerea*	He 6003	Hainan, China	–	MT248172	Xu et al. (2020)
*P. citrinosanguinea*	FP-105385	Massachusetts, United States	KP135100	KP135234	Floudas and Hibbett (2015)
*P. citrinosanguinea*	FD-287^*^	Massachusetts, United States	KP135095	–	Floudas and Hibbett (2015)
*P. citrinosanguinea*	FP-105385-Sp	United States	KP135100	KP135234	Floudas and Hibbett (2015)
*P. concrescens*	Spirin 7322	Russia	KP994380	KP994382	Volobuev et al. (2015)
*P. concrescens*	CHWC 1507-39	Taiwan, China	MZ422798	–	Chen et al. (2021)
*P. crystallina*	Chen 3576^*^	Taiwan, China	MZ422801	–	Chen et al. (2021)
*P. crystallina*	GC 1409-7	Taiwan, China	MZ422803	MZ637189	Chen et al. (2021)
*P. cumulodentata*	Wu 1708-91	Liaoning, China	MZ422804	MZ637190	Volobuev et al. (2015)
*P. cumulodentata*	LE 298935	Russia	KP994359	KP994386	Volobuev et al. (2015)
*P. cystidiata*	GC 1708-358^*^	Liaoning, China	LC412096	–	Wu et al. (2018a)
*P. cystidiata*	Wu 1708-326	Taiwan, China	LC412097	LC412100	Wu et al. (2018a)
*P. deflectens*	FCUG 2777	Turkey	–	GQ470644	Wu et al. (2010)
*P. ericina*	HHB-2288	United States	KP135167	KP135247	Floudas and Hibbett (2015)
*P. ericina*	HHB-2714	North Carolina, United States	KP135169	–	Floudas and Hibbett (2015)
*P. fusca*	Wu 1409-163	Hubei, China	LC412099	LC412106	Wu et al. (2018a)
*P. fusca*	Wu 1409-161^*^	Hubei, China	LC412098	LC412105	Wu et al. (2018a)
*P. fuscomarginata*	RLG-10834-Sp	New Mexico, United States	MZ422806	MZ637192	Chen et al. (2021)
*P. ginnsii*	Wu 9210-22^*^	Hubei, China	MZ422807	MZ637193	Chen et al. (2021)
*P. granulate*	GC 1703-5	Hubei, China	MZ422809	MZ637195	Chen et al. (2021)
*P. granulate*	Wu 9210-57^*^	Hubei, China	MZ422810	MZ637196	Chen et al. (2021)
*P. guangdongensis*	Wu 1809-348^*^	Guangdong, China	MZ422813	MZ637199	Chen et al. (2021)
*P. guangdongensis*	Wu 1809-359	Guangdong, China	MZ422814	MZ637200	Chen et al. (2021)
*P. hymenochaetoides*	He 5988^*^	Hainan, China	–	MT248173	Xu et al. (2020)
*P. incarnata*	WEI 16-075	Taiwan, China	MF399406	MF399397	Wu et al. (2018b)
*P. incarnata*	WEI 16-078^*^	Taiwan, China	MF399407	MF399398	Wu et al. (2018b)
*P. inflata*	Dai 10376	Jiangxi, China	JX623929	JX644062	Jia et al. (2014)
*P. krikophora*	GC 1602-73	Taiwan, China	MZ422816	MZ637202	Chen et al. (2021)
*P. krikophora*	HHB-6736-Sp	Florida, United States	MZ422817	MZ637203	Chen et al. (2021)
*P. laevis*	KHL11839	Sweden	EU118652	EU118652	Larsson (2007)
*P. laevis*	Wu 0309-40	Jilin, China	MZ422818	–	Chen et al. (2021)
*P. laevis*	HHB-15519	United States	KP135149	KP135249	Floudas and Hibbett (2015)
*P. leptocystidiata*	Dai 10468	Jiangxi, China	MT235684	MT248167	Xu et al. (2020)
*P. leptocystidiata*	He 5853^*^	Guangdong, China	MT235685	MT248168	Xu et al. (2020)
*P. livescens*	GC 1612-11	Taiwan, China	MZ422819	MZ637204	Floudas and Hibbett (2015)
*P. livescens*	FD-106	United States	KP135070	KP135253	Floudas and Hibbett (2015)
*P. magnoliae*	HHB-9829-Sp	United States	KP135089	KP135237	Floudas and Hibbett (2015)
*P. metuloidea*	He 2565^*^	Yunnan, China	–	MT248163	Xu et al. (2020)
*P. metuloidea*	He 2766	Yunnan, China	MT235682	MT248164	Xu et al. (2020)
*P. minor*	He 3977	Hainan, China	–	MT248169	Xu et al. (2020)
*P. minor*	He 3988^*^	Hainan, China	MT235686	MT248170	Xu et al. (2020)
*P. parmastoi*	WEI 16-481	Taiwan, China	MZ422822	MZ637207	Chen et al. (2021)
*P. parmastoi*	Wu 880313-6^*^	Taiwan, China	MZ422823	GQ470654	Chen et al. (2021)
*P. porostereoides*	He 1902	Shanxi, China	KX212217	KX212221	Liu and He (2016)
*P. pruinose*	CLZhao 7112	Yunnan, China	MZ435346	MZ435350	Wang and Zhao (2021)
*P. pruinose*	CLZhao 7113^*^	Yunnan, China	MZ435347	MZ435351	Wang and Zhao (2021)
*P. pseudomagnoliae*	PP-25	South Africa	KP135091	KP135250	Floudas and Hibbett (2015)
*P. pseudosanguinea*	FD-244	United States	KP135098	KP135251	Floudas and Hibbett (2015)
*P. queletii*	HHB-11463	Wisconsin, United States	KP134994	KP135235	Floudas and Hibbett (2015)
*P. queletii*	FP-102166	Illinois, United States	KP134995	–	Floudas and Hibbett (2015)
*P. rhizomorpha*	GC 1708-335^*^	Taiwan, China	MZ422824	MZ637208	Chen et al. (2021)
*P. rhizomorpha*	GC 1708-354	Taiwan, China	MZ422825	MZ637209	Chen et al. (2021)
*P. rhodella*	FD-18	United States	KP135187	KP135258	Floudas and Hibbett (2015)
*P. robusta*	Wu 1109-69	Jilin, China	MF399409	MF399400	Wu et al. (2018b)
*P. sanguinea*	HHB-7524	United States	KP135101	KP135244	Floudas and Hibbett (2015)
*P. sanguinea*	Niemela 7993	Finland	KP135105	–	Floudas and Hibbett (2015)
*P. sanguineocarnosa*	FD-359	United States	KP135122	KP135245	Floudas and Hibbett (2015)
***P. shenghuaii***	Dai 24610^*^	**Yunnan, China**	**OP874925**	**OP874920**	**Present study**
***P. shenghuaii***	**Dai 24609**	**Yunnan, China**	**OP874924**	**OP874919**	**Present study**
*P. sinensis*	GC 1809-56	Taiwan, China	MT235689	MT248176	Xu et al. (2020)
*P. sinensis*	He 4660^*^	Liaoning, China	MT235688	MT248175	Xu et al. (2020)
*P. sordida*	FD-241	United States	KP135136	KP135252	Floudas and Hibbett (2015)
*Phanerochaete s.l.* sp.	TJV-93-262-T	Louisiana, United States	KP135021	–	Floudas and Hibbett (2015)
*Phanerochaete s.l.* sp.	RLG-13408-Sp	Louisiana, United States	KP135020	–	Floudas and Hibbett (2015)
*Phanerochaete* sp.	FCUG 2777	Turkey	MZ422830	–	Wu et al. (2010)
*P. spadicea*	Wu 0504-11	Yunnan, China	MZ422836	–	Chen et al. (2021)
*P. spadicea*	Wu 0504-15^*^	Yunnan, China	MZ422837	–	Chen et al. (2021)
*P. stereoides*	He 2309	Hunan, China	KX212219	KX212223	Liu and He (2016)
*P. subceracea*	FP-105974-R	United States	KP135162	KP135255	Floudas and Hibbett (2015)
*P. subrosea*	He 2421^*^	Ningxia, China	MT235687	MT248174	Xu et al. (2020)
*P. taiwaniana*	Wu 880824-17^*^	Taiwan, China	MZ422842	GQ470666	Chen et al. (2021)
*P. taiwaniana*	Wu 0112-13	Taiwan, China	MF399412	MF399403	Wu et al. (2018b)
*P. thailandica*	2015-07^*^	Thailand	MF467737	–	Chen et al. (2021)
*P. thailandica*	Wu 1710-3	Vietnam	MZ422843	MZ637223	Chen et al. (2021)
*P. velutina*	Kotiranta 25567	Russia	KP994354	KP994387	Volobuev et al. (2015)
*P. xerophila*	HHB-8509-Sp	Arizona, United States	KP134996	KP135259	Floudas and Hibbett (2015)
*P. xerophila*	KKN-172	Arizona, United States	KP134997	–	Floudas and Hibbett (2015)
*P. yunnanensis*	He 2697	Yunnan, China	–	MT248165	Xu et al. (2020)
*P. yunnanensis*	He 2719^*^	Yunnan, China	MT235683	MT248166	Xu et al. (2020)
*Phlebiopsis brunneocystidiata*	Chen 666	Taiwan, China	MT561707	GQ470640	Wu et al. (2010)
*P. crassa*	He 5205	Vietnam	MT452523	MT447448	Zhao et al. (2021)
*P. cylindrospora*	He 5984^*^	Hainan, China	MT386404	MT447445	Zhao et al. (2021)
*P. friesii*	He 5820	Sri Lanka	MT452530	MT447415	Zhao et al. (2021)
*P. magnicystidiata*	He 5648^*^	Hunan, China	MT386377	MT447409	Zhao et al. (2021)
*P. membranacea*	He 3849^*^	Hainan, China	MT386401	MT447441	Zhao et al. (2021)
*P. sinensis*	He 4673^*^	Sichuan, China	MT386397	MT447435	Zhao et al. (2021)
*P. yunnanensis*	CLZhao 3990	Yunnan, China	MH744141	MH744143	Zhao et al. (2019)
*Rhizochaete americana*	FP-102188	Illinois, United States	KP135409	KP135277	Floudas and Hibbett (2015)
*R. americana*	HHB2004	Georgia, United States	AY219391	AY219391	Greslebin et al. (2004)
*R. belizensis*	FP150712	Belize	KP135408	KP135280	Floudas and Hibbett (2015)
*R. borneensis*	WEI16-426	Taiwan, China	MZ637070	MZ637270	Chen et al. (2021)
*R. brunnea*	MR11455	Argentina	AY219389	AY219389	Greslebin et al. (2004)
*R. filamentosa*	FP105240	Indiana, United States	KP135411	AY219393	Nakasone et al. (2017)
*R. filamentosa*	HHB 3169	Maryland, United States	KP135410	KP135278	Floudas and Hibbett (2015)
*R. fissurata*	CLZhao2200	Yunnan, China	MZ713640	MZ713844	Gu and Zhao (2021)
*R. fissurata*	CLZhao7965	Yunnan, China	MZ713641	MZ713845	Gu and Zhao (2021)
*R. fissurata*	CLZhao10407^*^	Yunnan, China	MZ713642	MZ713846	Gu and Zhao (2021)
*R. fissurata*	CLZhao10418	Yunnan, China	MZ713643	MZ713847	Gu and Zhao (2021)
*R. flava*	PR 1141	Puerto Rico	KY273030	KY273033	Nakasone et al. (2017)
*R. flava*	PR3148	Puerto Rico	KY273029	–	Nakasone et al. (2017)
*R. fouquieriae*	KKN-121	Arizona, United States	AY219390	GU187608	Nakasone et al. (2017)
*R. fouquieriae*	KKN-121sp	United States	KY948786	KY948858	Justo et al. (2017)
*R. grandinosa*	CLZhao3117^*^	Yunnan, China	MZ713644	MZ713848	Gu and Zhao (2021)
*R. lutea*	Wu 880417-5	Taiwan, China	MZ637072	GQ470651	Chen et al. (2021)
*R. radicata*	FD123	Massachusetts, United States	KP135407	KP135279	Floudas and Hibbett (2015)
*R. radicata*	FD338	Massachusetts, United States	KP135406	–	Floudas and Hibbett (2015)
*R. radicata*	HHB1909	Highlands, United States	AY219392	AY219392	Greslebin et al. (2004)
*R. rubescens*	Wu0910-45	Beijing, China	LC387335	MF110294	Chen et al. (2018)
*R. sulphurina*	DLL2014-176	Idaho, United States	KY273032	–	Nakasone et al. (2017)
*R. sulphurina*	HHB5604	Montana, United States	KY273031	GU187610	Nakasone et al. (2017)
*R. sulphurina*	KHL16087	Brazil	KT003523	–	Chikowski et al. (2016)
*R. sulphurina*	URM87190	Brazil	KT003522	KT003519	Chikowski et al. (2016)
***R. variegata***	Dai 24600^*^	**Guizhou, China**	**OP874926**	**OP874921**	**Present study**
***R. variegata***	**Dai 24601**	**Guizhou, China**	**OP874927**	**OP874922**	**Present study**

New species are in bold with type specimens marked with an asterisk (*).

### Phylogenetic analyses

New sequences, deposited in GenBank (http://www.ncbi.nlm.nih.gov/genbank/) (**Table 1**), were aligned with additional sequences retrieved from GenBank (**Table 1**) using BioEdit 7.0.5.3 (Hall, 1999) and ClustalX 1.83 (Thompson et al., 1997), followed by manual adjustment. Sequence alignment was deposited at TreeBase (http://purl.org/phylo/treebase/; submission ID 29897). Sequences of *Bjerkandera adusta* (Willd.) P. Karst. and *B. centroamericana* Kout et al. were used as outgroups (Chen et al., 2021). Maximum likelihood (ML) and Bayesian inference (BI) methods were used for the phylogenetic analysis. The GTR + I + G model was estimated as the best-fit evolutionary model by PhyloSuite 1.2.2 (Zhang et al., 2020) using the Akaike information criterion. The ML analysis was carried out with RAxML 8.2.12 (Stamatakis, 2006; Silvestro and Michalak, 2012), and the BI tree reconstruction was carried out with MrBayes 3.2.5 (Ronquist et al., 2012). Four Markov chains were run for two runs from random starting trees for 10 million generations, and trees were sampled every 1,000 generations. The burn-in was set to discard 25% of the trees. A majority rule consensus tree of all the remaining trees was calculated. Branches that received bootstrap support for ML and Bayesian posterior probabilities (BPP) greater than or equal to 75% (ML) and 0.95 (BPP) were considered as significantly supported.

## Results

### Phylogeny

The ITS + nLSU dataset included 155 fungal collections representing 101 taxa of the family Phanerochaetaceae. PhyloSuite suggested GTR + I + G to be the best-fit models of nucleotide evolution for BI. Bayesian analysis resulted in a concordant topology with an average standard deviation of split frequencies = 0.006701. The ML and BI analyses resulted in nearly identical topologies, and thus, only the ML tree is presented with the ML and BPP when they were greater than or equal to 50% and 0.90, respectively.

The phylogram inferred from ITS + nLSU sequences within the family Phanerochaetaceae highlighted two undescribed species nested in *Phanerochaete* and *Rhizochaete*, respectively. *Phanerochaete shenghuaii* formed an independent lineage with a robust support (ML = 99, BPP = 1.0) and stably nested within the core *Phanerochaete* clade. *Rhizochaete variegata* clustered in *Rhizochaete* clade with high support (ML = 99, BPP = 1.0) and grouped with *Rhizochaete radicata* (Henn.) Gresl. et al. and *R. grandinosa* C.L. Zhao & Z.R. Gu.

### Taxonomy

**Figure 1:**
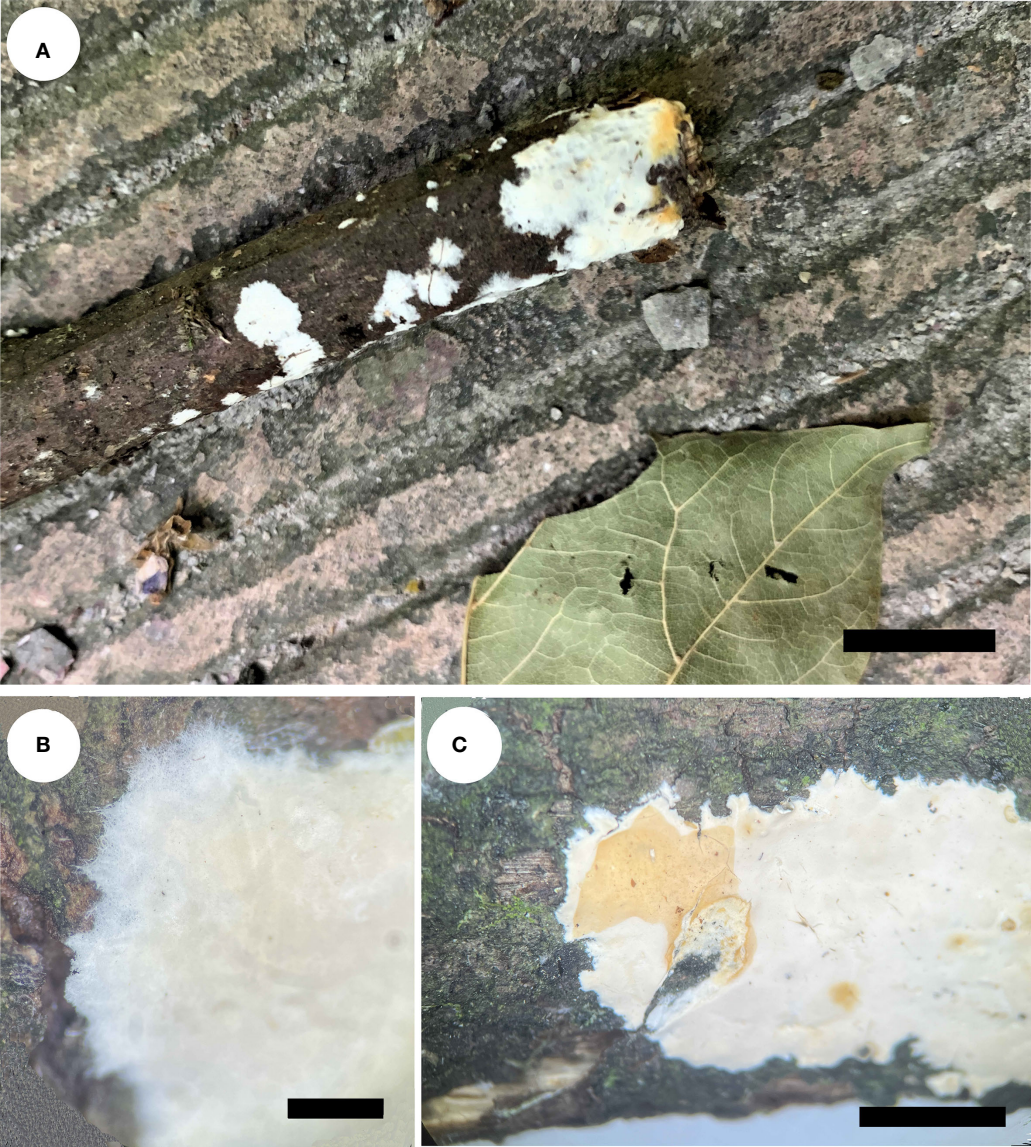
Basidiocarps of *Phanerochaete shenghuaii* (holotype, Dai 24610). **(A)** *In situ*. **(B)** Detailed view of the margin. **(C)** Reaction with KOH. Scale bars: **(A)** = 1 cm, **(B)** = 2 mm, **(C)** = 0.5 cm.

**Figure 2:**
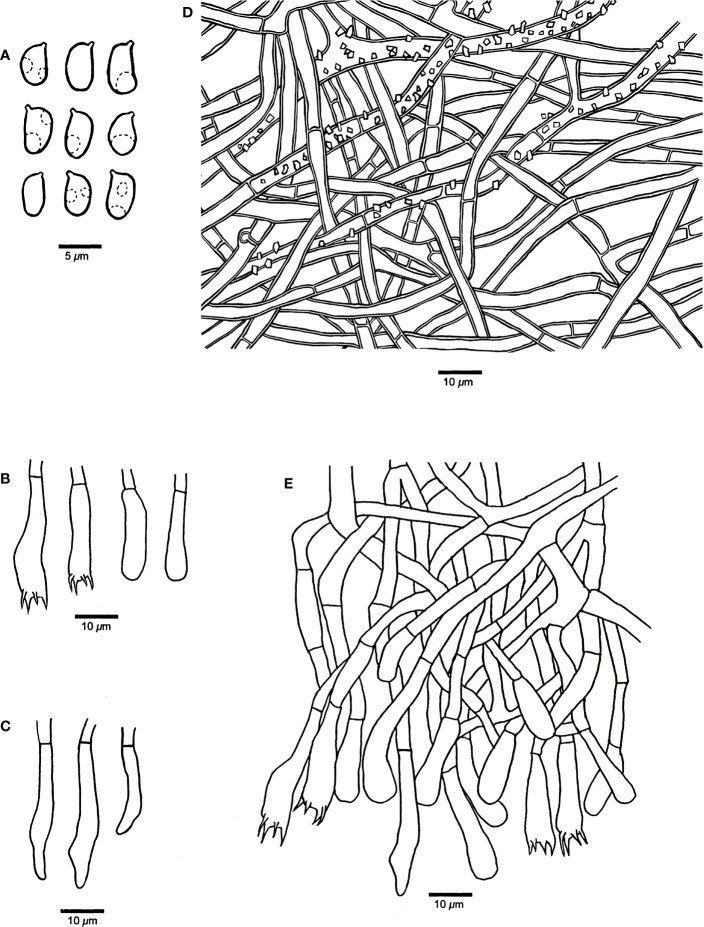
Microscopic structures of *Phanerochaete shenghuaii* (drawn from the holotype, Dai 24610). **(A)** Basidiospores. **(B)** Basidia and basidioles. **(C)** Cystidia. **(D)** A vertical section of the subiculum. **(E)** A vertical section of the hymenium.

*Phanerochaete shenghuaii* Q.Y. Zhang, Y.C. Dai & Jing Si, sp. nov., **Figures 1**, **2**

MycoBank: 847200

*Type* — China, Yunnan Province, Zhaotong, Daguan County, Huanglianhe Scenic Spot, on fallen liana branch, 16 July 2022, Dai 24610 (holotype, BJFC038931).

*Etymology* — *Shenghuaii* (Lat.): In honor of Professor Sheng-Hua Wu, the Chinese mycologist.

*Basidiocarps* — Annual, effused, adnate, inseparable from substrate, membranaceous to subceraceous, up to 2.5 cm long, 1.5 cm wide, and 0.2 mm thick in section. Hymenial surface ivory white to cream when juvenile, buff to yellowish brown with age, buff in KOH, smooth, uncracked; margin concolorous with hymenial surface, thinning out, usually rhizomorphic.

*Hyphal structure* — Hyphal system monomitic; generative hyphae mostly simple septate, occasionally with clamp connections in subiculum, IKI−, CB−; tissue unchanged in KOH.

*Subiculum* — Subicular hyphae hyaline, slightly thick-walled, frequently simple septate, occasionally with clamp connections, frequently branched, usually strongly encrusted with crystal granules, interwoven, 3–5 μm in diameter.

*Hymenophore* — Subhymenial hyphae hyaline, thin-walled, smooth, simple septate, frequently branched, interwoven, 2.5–5 μm in diameter; cystidia smooth, immersed or projecting from hymenium, narrowly fusiform or clavate with pointed tips, hyaline, thin-walled, smooth, with a simple septum at the base, 18–35 × 3–5 µm; basidia clavate, with a basal simple septum and four sterigmata, 22–30 × 4–5 µm; basidioles similar to basidia in shape, but slightly smaller.

*Basidiospores* — Ellipsoid with a distinct apiculus, hyaline, thin-walled, smooth, occasionally with one or two guttules, IKI−, CB−, (4.5–)4.8–6(–6.4) × 2.5–3.8(–4) µm, *L* = 5.26 µm, *W* = 3.01 µm, *Q* = 1.71–1.79 (*n* = 60/2).

Additional specimen (paratype) examined — China, Yunnan Province, Zhaotong, Daguan County, Huanglianhe Scenic Spot, on fallen angiosperm branch, 16 July 2022, Dai 24609 (BJFC038930).

**Figure 3:**
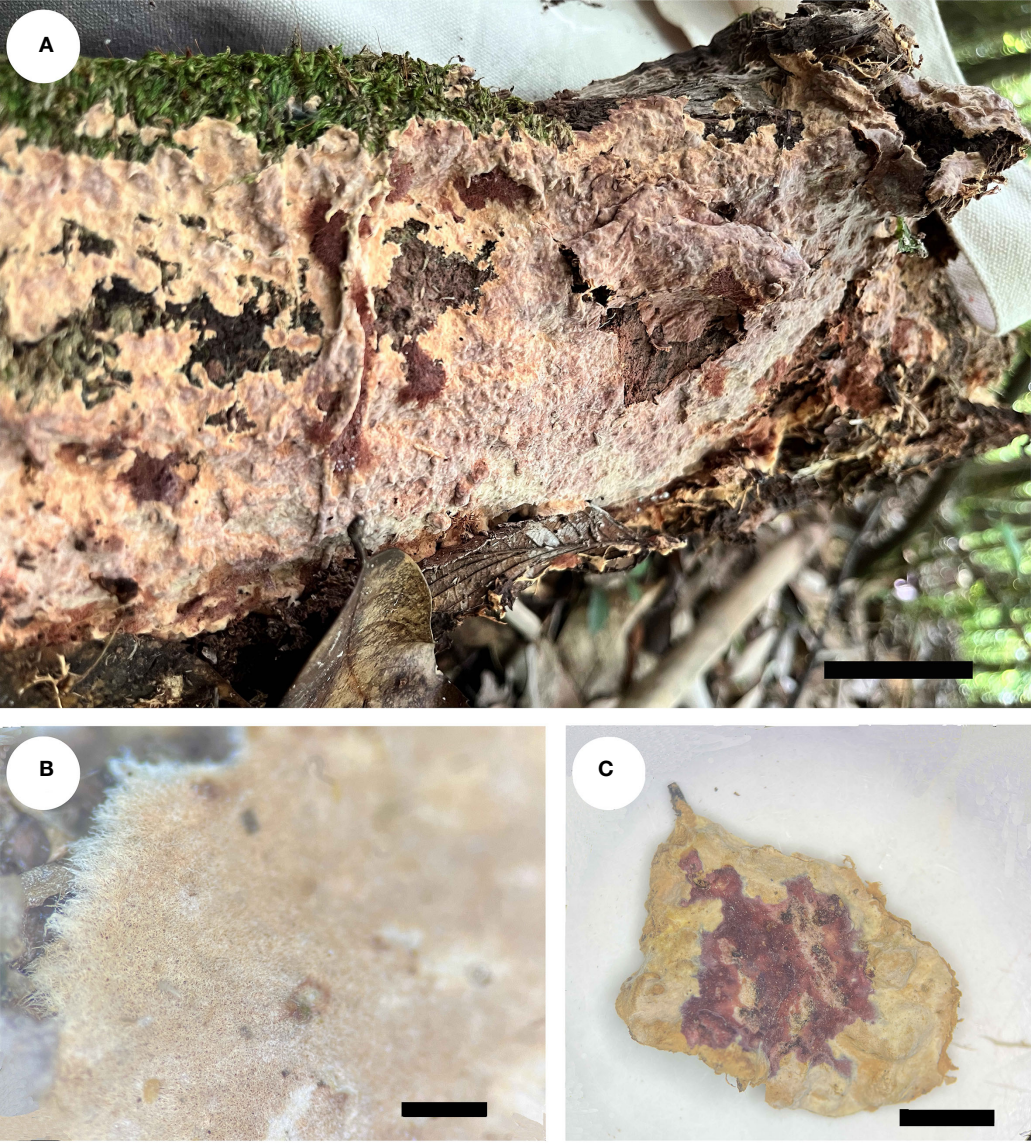
Basidiocarps of *Rhizochaete variegata* (holotype, Dai 24600). **(A)** *In situ*. **(B)** Detailed view of the margin. **(C)** Reaction with KOH. Scale bars: **(A)** = 1 cm, **(B)** = 1 mm, **(C)** = 0.5 cm.

**Figure 4:**
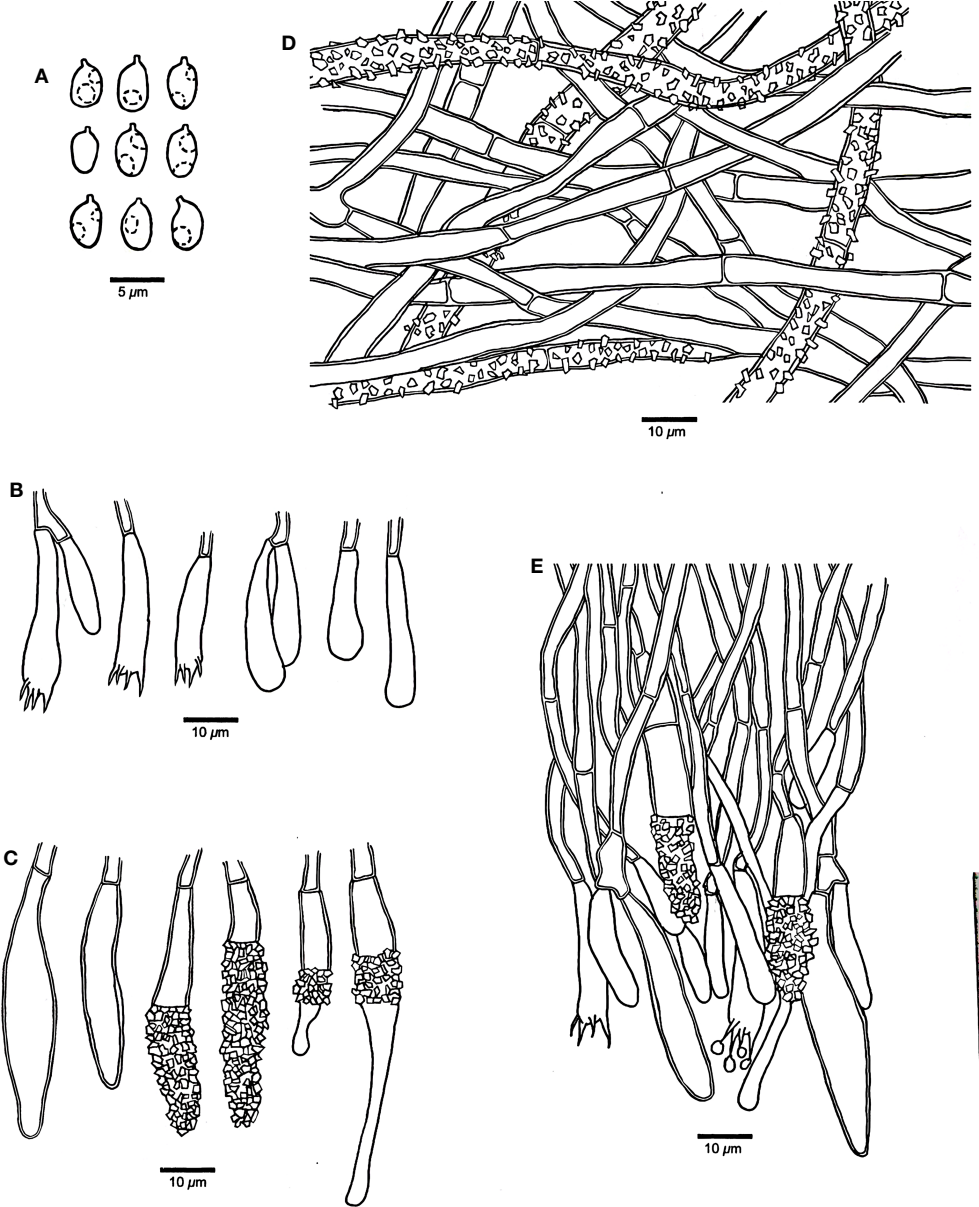
Microscopic structures of *Rhizochaete variegata* (drawn from the holotype, Dai 24600). **(A)** Basidiospores. **(B)** Basidia and basidioles. **(C)** Cystidia. **(D)** A vertical section of the subiculum. **(E)** A vertical section of the hymenium.

*Rhizochaete variegata* Q.Y. Zhang, Y.C. Dai & Jing Si, sp. nov., **Figures 3**, **4**

MycoBank: 847201

*Type* — China, Guizhou Province, Zunyi, Suiyang County, Kuankuoshui Nature Reserve, on fallen angiosperm trunk, 07 July 2022, Dai 24600 (holotype, BJFC038928).

*Etymology* — *Variegata* (Lat.): referring to the species having variable cystidia.

*Basidiocarps* — Annual, effused, loosely adnate, easily separable from substrate, membranaceous, soft, fragile, up to 9 cm long, 3.5 cm wide, and 1 mm thick in section. Hymenial surface buff-yellow to clay-pink when fresh, cream to buff upon drying, violet in KOH, smooth or locally tuberculate, occasionally cracked; margin darker or concolorous with hymenial surface, thinning out, usually rhizomorphic.

*Hyphal structure* — Hyphal system monomitic; generative hyphae simple septate, IKI−, CB−; tissue unchanged in KOH.

*Subiculum* — Subicular hyphae hyaline, slightly thick-walled, simple septate, rarely branched, bearing abundant crystal granules, strongly interwoven, 3.5–6 μm in diameter.

*Hymenophore* — Subhymenial hyphae hyaline, slightly thick-walled, smooth, simple septate, more or less regularly arranged, 3–5 μm in diameter. Hymenium contains a dense palisade of cystidia and basidia, IKI−, CB−; cystidia numerous, immersed or projecting from hymenium, clavate, subfusiform or subulate with an obtuse apex, hyaline, slightly thick-walled, some with thin-walled apex, with a simple septum at the base, some apically or centrally encrusted, 28–52 × 5–8 µm; basidia narrowly clavate, with a basal simple septum and four sterigmata, 30–45 × 4–5 µm; basidioles similar to basidia in shape, but slightly smaller.

*Basidiospores* — Ellipsoid with a distinct apiculus, hyaline, thin-walled, smooth, occasionally with one or two small guttules, IKI−, CB−, 3–4(–4.2) × (2–)2.2–3(–3.2) µm, *L* = 3.61 µm, *W* = 2.72 µm, *Q* = 1.27–1.38 (*n* = 60/2).

Additional specimen (paratype) examined — China, Guizhou Province, Zunyi, Suiyang County, Kuankuoshui Nature Reserve, on fallen angiosperm trunk, 07 July 2022, Dai 24601 (BJFC038929).

## Discussion

Southwest China has a complex topography and geography, luxuriant vegetation, and virgin forests and has highly variable weather including tropical, subtropical, and alpine climates, thus providing a favorable region for the growth and reproduction of higher fungi (Yuan and Dai, 2008; Dai et al., 2021; Wu et al., 2022a). The extremely high fungal diversity in this area has attracted much attention from mycologists both at home and abroad (Feng and Yang, 2018). It is worth noting that the two new corticioid species *P. shenghuaii* and *R. variegata* were collected from Northeast Yunnan and Northwest Guizhou, respectively, and the type locality of the two new species is in a typical subtropical climate.

*Phanerochaete shenghuaii* is characterized by white to cream basidiocarps with rhizomorphic margin, encrusted subicular hyphae, and smooth cystidia. Morphologically, three species, *Phanerochaete rhizomorpha* C.C. Chen et al., *P. leptocystidiata* Y.L. Xu & S.H. He, and *P. sinensis* Y.L. Xu et al., are similar to *P. shenghuaii* by sharing similar basidiocarps, rhizomorphic margin, and smooth cystidia. However, *P. rhizomorpha* is described from Taiwan Province, China, and differs from *P. shenghuaii* by its subcapitate to cylindrical cystidia with obtuse apices and smaller basidiospores (3.9–5.3 × 2.1–3 μm vs. 4.8–6 × 2.5–3.8 µm, Chen et al., 2021). *Phanerochaete leptocystidiata* is widely distributed in South China and differs from *P. shenghuaii* by its basidiocarps easily separable from substrate and longer cystidia (30–70 μm in length vs. 18–35 µm in length, Xu et al., 2020). *Phanerochaete sinensis* is distinguished from *P. shenghuaii* in having longer cystidia (35–50 µm in length vs. 18–35 µm in length) and smaller basidiospores (4–5 × 2–2.5 μm vs. 4.8–6 × 2.5–3.8 µm, Xu et al., 2020).

In addition, the diversity of flora of seed plants and the distinctly diverse climates in Yunnan Province both contribute to the suitable substrates and environments for *Phanerochaete* species. Recently, a large number of *Phanerochaete* species have been found in Yunnan Province (Xiong and Dai, 2009; Wu et al., 2010; Xu et al., 2020; Chen et al., 2021; Wang and Zhao, 2021). Among them, *Phanerochaete yunnanensis* Y.L. Xu & S.H. He is similar to *P. shenghuaii* by growing on dead liana and fallen angiosperm branches but differs by grandinioid basidiocarps and the absence of cystidia. *Phanerochaete pruinose* C.L. Zhao and D.Q. Wang is similar to *P. shenghuaii* by sharing white and smooth hymenophore, but differs by lacking cystidia and having thinner basidiospores (1.5–2.7 μm in width vs. 2.5–3.8 µm in width, Wang and Zhao, 2021). It is still noteworthy that *P. rhizomorpha* C.L. Zhao and D.Q. Wang described from Yunnan Province is an invalid name, attributed to the priority of *P. rhizomorpha* C.C. Chen et al. (Chen et al., 2021; Wang and Zhao, 2021). In addition, the two taxa represent two independent species according to their distinctive DNA sequences and morphology.

**Figure 5:**
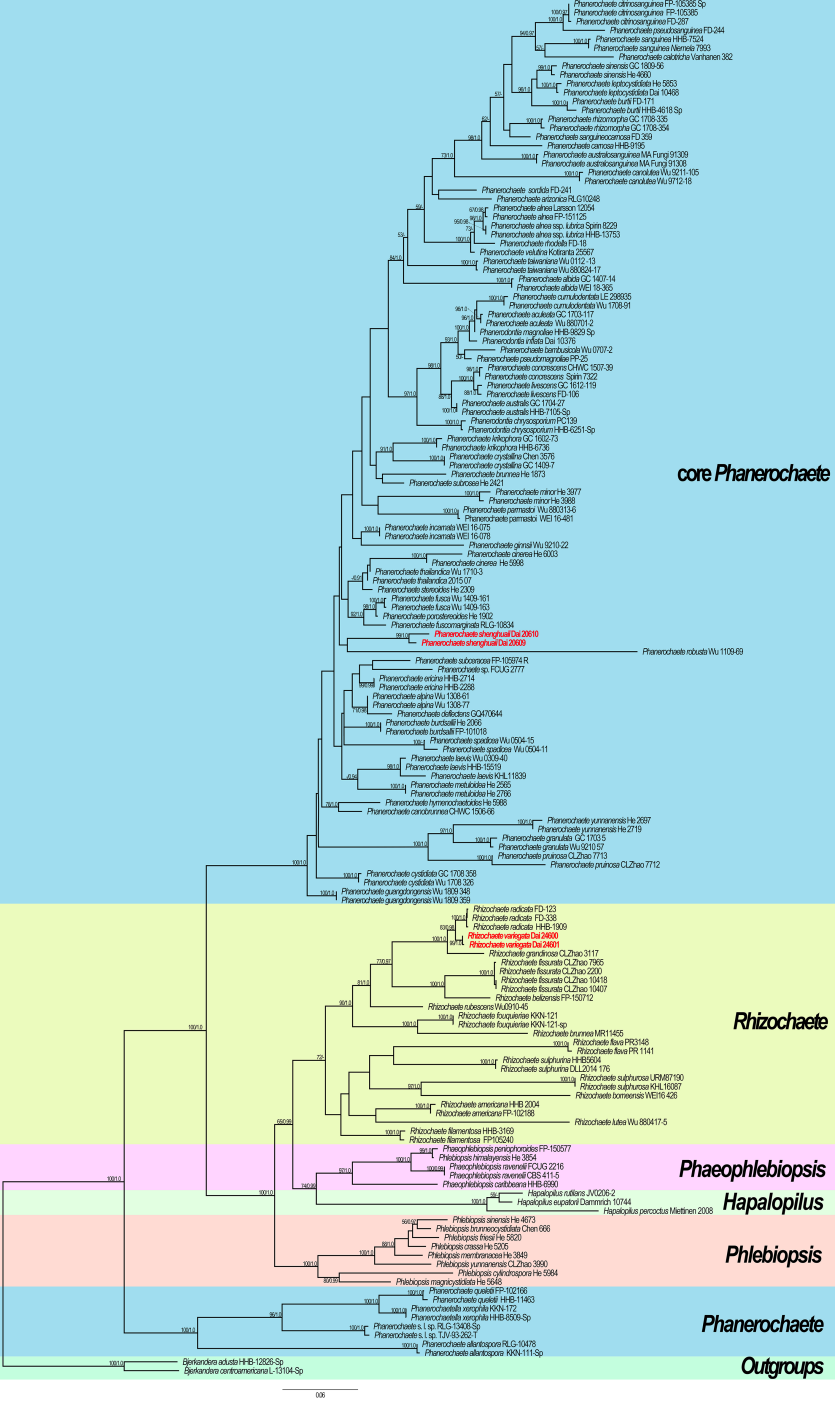
Maximum likelihood (ML) tree illustrating the phylogeny of the two new species in *Phanerochaetaceae* based on ITS + nLSU sequences. Branches are labeled with ML bootstrap >50% and Bayesian posterior probabilities (BPP) >0.90, respectively. New species are highlighted by red text.

Our phylogenetic analysis demonstrates that *Rhizochaete* is monophyletic with a low support and clusters as a sister clade to *Hapalopilus*, *Phaeophlebiopsis*, and *Phlebiopsis*. Two specimens of *R. variegata* form a lineage with a strong support (ML = 99, BPP = 1.0, **Figure 5**). *Rhizochaete variegata* is closely related to *R. grandinosa* and *R. radicata* (ML = 100, BPP = 1, **Figure 5**), and these three species share curry-yellow hymenial surface, violet in KOH, thick-walled and encrusted subicular hyphae, and similar-sized basidiospores. However, *R. variegata* has abundant variable and slightly thick-walled cystidia with a thin-walled apex, which can be readily distinguished from *R. grandinosa* and *R. radicata* (Greslebin et al., 2004; Gu and Zhao, 2021). Furthermore, there are differences of more than eight base pairs between their sequences, which amounts to 2% nucleotides in the ITS regions. Morphologically, *Rhizochaete sulphurosa* (Bres.) Chikowski et al. may be confused with *R. variegata* by sharing yellow basidiocarps, hymenial surface violet in KOH, and thin or slightly thick-walled (<1 µm) cystidia. Nevertheless, *R. sulphurosa* differs from *R. variegata* by its longer basidiospores (4.5–5.5 µm in length vs. 3–4 µm in length, Chikowski et al., 2016).

Although more taxa of Phanerochaetaceae have been described (Greslebin et al., 2004; Bianchinotti et al., 2005; Chen et al., 2021), the taxonomy of corticioid fungi in Polyporales is woefully understudied. Many closely related genera are difficult to differentiate based on apparently plesiomorphic morphology, such as *Phanerochaete*, *Rhizochaete*, *Phaeophlebiopsis*, and *Hapalopilus* (Bianchinotti et al., 2005; Chen et al., 2021). *Rhizochaete* is separated from *Phanerochaete* mainly by their basidiocarp reaction with KOH (Greslebin et al., 2004; Chen et al., 2021). Indeed, this character is delimited in most species of the two genera. However, there are still some species of *Phanerochaete* exhibiting red in KOH, such as *P. affinis* (Burt) Parmasto and *P. aurantiobadia* Ghob.-Nejh. et al. (Punugu et al., 1980; Ghobad-Nejhad et al., 2015). Therefore, more samples from worldwide and multigene phylogeny analysis are urgently needed for understanding the diversity of corticioid species of Polyporales.

Southwest China is a hotspot for fungal diversity, and numerous taxa of wood-inhabiting fungi have been described from this area based on morphological and molecular phylogenetic analyses (Dai, 2010; Zhao et al., 2015; Zhou et al., 2016; Dai et al., 2021; Guan and Zhao, 2021; Wang et al., 2021; Wu et al., 2021; Wu et al., 2022b). Notably, the species diversity of corticioid fungi in this area is still not well-known, and therefore, the present paper confirms that more unknown species exist in this area.

## Data availability statement

The datasets presented in this study can be found in online repositories. The names of the repository/repositories and accession number(s) can be found below: https://www.ncbi.nlm.nih.gov/genbank/, OP874919, OP874920, OP874921, OP874922, OP874924, OP874925, OP874926, OP874927.

## Author contributions

Q-YZ, Z-BL, and JS designed the research and contributed to data analysis and interpretation. Q-YZ prepared the samples and drafted the manuscript. Z-BL conducted molecular experiments and analyzed the data. H-GL and JS discussed the results and edited the manuscript. All authors contributed to the article and approved the submitted version.
